# Sex Differences, Genetic and Environmental Influences on Dilated Cardiomyopathy

**DOI:** 10.3390/jcm10112289

**Published:** 2021-05-25

**Authors:** Angita Jain, Nadine Norton, Katelyn A. Bruno, Leslie T. Cooper, Paldeep S. Atwal, DeLisa Fairweather

**Affiliations:** 1 Department of Cardiovascular Medicine, Mayo Clinic, 4500 San Pablo Road, Jacksonville, FL 32224, USA; jain.angita@mayo.edu (A.J.); bruno.katelyn@mayo.edu (K.A.B.); cooper.leslie@mayo.edu (L.T.C.J.); 2 Department of Cancer Biology, Mayo Clinic, 4500 San Pablo Road, Jacksonville, FL 32224, USA; norton.nadine@mayo.edu; 3 Genomic and Personalized Medicine, Atwal Clinic, 214 Brazilian Avenue, Suite 230, Palm Beach, FL 33480, USA

**Keywords:** dilated cardiomyopathy, familial dilated cardiomyopathy, idiopathic dilated cardiomyopathy, sex differences, sex ratio, pathogenesis, environment, virus, genes

## Abstract

Dilated cardiomyopathy (DCM) is characterized by dilatation of the left ventricle and impaired systolic function and is the second most common cause of heart failure after coronary heart disease. The etiology of DCM is diverse including genetic pathogenic variants, infection, inflammation, autoimmune diseases, exposure to chemicals/toxins as well as endocrine and neuromuscular causes. DCM is inherited in 20–50% of cases where more than 30 genes have been implicated in the development of DCM with pathogenic variants in *TTN* (Titin) most frequently associated with disease. Even though male sex is a risk factor for heart failure, few studies have examined sex differences in the pathogenesis of DCM. We searched the literature for studies examining idiopathic or familial/genetic DCM that reported data by sex in order to determine the sex ratio of disease. We found 31 studies that reported data by sex for non-genetic DCM with an average overall sex ratio of 2.5:1 male to female and 7 studies for familial/genetic DCM with an overall average sex ratio of 1.7:1 male to female. No manuscripts that we found had more females than males in their studies. We describe basic and clinical research findings that may explain the increase in DCM in males over females based on sex differences in basic physiology and the immune and fibrotic response to damage caused by mutations, infections, chemotherapy agents and autoimmune responses.

## 1. Introduction

Dilated cardiomyopathy (DCM) is characterized by dilatation of the left ventricle and impaired systolic function. The American Heart Association classifies DCM as genetic, mixed or acquired, whereas the European Society of Cardiology classifies DCM as genetic (familial) or non-genetic (non-familial) [1]. The World Health Organization (WHO) defines DCM as a serious cardiac disorder in which structural or functional abnormalities of the heart muscle can lead to substantial morbidity and mortality owing to complications such as heart failure and arrhythmia [1]. DCM is the second most common cause of heart failure after coronary heart disease, with an estimated prevalence of 1 in 250–500 people [2]. The etiology of DCM is diverse including pathogenic genetic variants, infection, inflammation, autoimmune diseases, exposure to toxins as well as endocrine or neuromuscular causes [3]. DCM presents between the ages of 20–60 but can occur in children or the elderly. DCM often has a long latency period and patients can be clinically asymptomatic.

After identifying systolic dysfunction and a morphologically enlarged left ventricle, an etiological assessment is undertaken and, if no identifiable cause (except genetic) can be found, a clinical diagnosis of ‘idiopathic DCM’ is assigned [3]. Idiopathic cardiomyopathy is likely influenced by an individual’s genetic profile and environmental factors. If an individual had DCM from an environmental factor, like chemotherapy, their DCM would not be considered to be idiopathic. DCM is considered to be familial when more than one first-degree relative has been diagnosed with DCM or has had sudden cardiac death at a young age [2]. DCM is inherited in 20–50% of cases and abnormalities are seen frequently on echocardiogram in asymptomatic relatives [3]. Inheritance of idiopathic DCM is primarily autosomal dominant, although other modes of inheritance have been observed. DCM can also occur secondary to or in conjunction with systemic disease or syndromes. Determining the precise molecular etiology responsible for familial DCM impacts medical management and allows early identification of those at increased risk [3]. In contrast to many Mendelian disorders, genetic associations present in familial cardiomyopathies have variable penetrance, and manifestations might also result from multiple low penetrance alleles or additional genes with a modifying (perhaps protective) effect on severity or age of onset, or when exposure to an additional insult occurs [4]. We also note the existence of sporadic cases of DCM that may result from multiple low penetrance alleles within the population [5]. The identification of the pathogenic variant(s) might have important complex trait predictive and therapeutic implications.

## 2. Sex Differences

Sex-specific differences in genes are known to exist in the hearts of healthy men and women [6,7]. Male sex is an important risk factor for developing heart failure in a number of cardiovascular conditions including DCM [8,9]; however, few clinical studies have examined sex differences in incidence or pathogenetic mechanisms in DCM specifically. One study found that men with DCM had more apoptosis-related protein expression compared to women [10]. Additionally, men with myocarditis/acute DCM present with almost a twofold greater incidence of myocardial fibrosis compared to women using cardiac MRI [11,12], with fibrosis being implicated as an important factor in the pathogenesis of DCM. Sex differences observed in DCM have been attributed primarily to the effect of sex hormones on cardiac resident cells and cardiac inflammation [13] and multiple mouse models lacking estrogen receptors have demonstrated that estrogen improves cardiac function [14,15,16]. The major steroid hormones that have been studied in cardiovascular diseases and the immune response to cardiac damage or infection are estrogens, testosterone and progesterone. These so-called sex hormones bind to nuclear-associated receptors in cardiac cells like cardiomyocytes and fibroblasts where they influence gene expression. Estrogen receptor alpha (ERα) and beta (ERβ) and the androgen receptor (AR) are sequestered in the cytoplasm bound to heat shock proteins. When activated by ligand, they bind directly to estrogen response elements (EREs) or androgen response elements (ARE) in the promoter region of specific genes or indirectly activate gene transcription by binding transcription factors [17,18]. Many of the genes that are influenced by estrogen/ERE in the heart are known to promote reparative remodeling rather than fibrosis, which is a primary factor that leads to dilatation and heart failure.

Whenever damage occurs to the heart from genetic and/or environmental causes, immune cells are recruited to the heart. Sex steroids alter immune cell function via nuclear and non-nuclear membrane expression of hormone receptors. Signaling through membrane hormone receptors is more rapid than nuclear receptors and can result in both gene transcription through initiation of kinase signal pathways or in non-transcriptional signals via calcium flux and activation of glutamate receptors [18,19,20]. The mechanisms of how estrogen protects against cardiomyopathy are not fully understood, but mouse models of cardiomyopathy lacking the estrogen receptor show increased expression of cardiac calcium channels [21].

**Figure 1:**
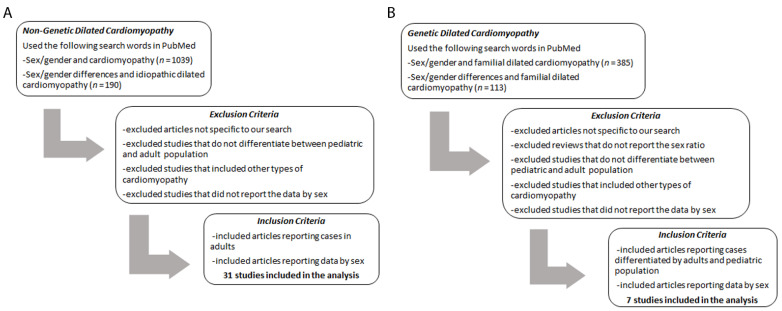
Study Flowchart. (**A**) Non-genetic dilated cardiomyopathy vs. (**B**) genetic dilated cardiomyopathy.

The American Heart Association scientific statement on DCM published in 2016 concluded that the overall effect of female sex on heart failure, and DCM especially, was not clear at that time and that women may be underrepresented in clinical trials [22] as is observed from registries found in Table 1 that show fewer women than men in studies of DCM. Even though it is quite likely that women are underrepresented in clinical trials of DCM, another explanation for the observation is that the number of men and women recruited to studies generally represents a sex difference in presentation of the disease similar to what has been found to occur for other cardiovascular and autoimmune diseases [23,24]. The EuroHeart Failure Survey II found that DCM occurred more often in males than females [25]. An estimate of DCM prevalence was derived from a population-based study conducted in Olmstead County, Minnesota that reported a male to female sex ratio of 3:1 [26]. However, there is a lack of data in the literature on whether sex differences exist in phenotype, severity and/or outcomes for most epidemiological studies of DCM [25,27,28]. For this review, we queried PubMed using the search terms “sex/gender and cardiomyopathy” and “sex/gender differences and idiopathic dilated cardiomyopathy” to identify studies reporting DCM by sex. We found 1229 studies in our search; however, many studies were rejected for reasons that included the sex not being reported in the study, which was a frequent finding in our search, and the study not distinguishing the type of cardiomyopathy (Figure 1). Additionally, we did not include types of DCM in this analysis that occur in only one sex such as peripartum cardiomyopathy.

After exclusions, 31 studies were selected for the analysis of sex ratios in DCM from all causes (Table 1, Figure 1A). Table 1 includes data from adults with DCM from all causes such as viral and idiopathic DCM. The largest study from our search that reported data by sex was the United Network of Organ Sharing (UNOS) registry database, which identified 16,091 patients with DCM where 11,059 were males and 5032 females—a sex ratio of 2.2:1 men to women (Table 1).

The multinational cardiomyopathy registry of the EURObservational Research Programme is a prospective, observational study published in 2018 comprised of 1260 patients with DCM out of a total of 3208 patients [55]. They examined 935 males and 325 females in their study, which is a sex ratio of 2.8:1 (Table 1). A study of 881 patients with DCM where 591 were males and 290 females indicated a sex ratio of 2:1 men to women [53]. Another study with 853 patients with DCM included 614 males and 239 females, which makes a sex ratio of 2.5:1 men to women [49]. We highlight these studies because they have a larger sample size than the other studies listed in Table 1. It is important to emphasize that these studies were not designed to examine sex differences and the number of men and women simply reflect the number of patients recruited to the registries or clinical studies. However, even with this caveat these data are the best indicator of the sex differences in DCM. Of note, none of the studies reported more women than men with DCM in the study. Overall, the five studies with numbers of patients with DCM over 500 from our search (bold in Table 1) generated an average sex ratio of 2.4:1 men to women. The overall combined average sex ratio for DCM in adults from all 31 studies combined was 2.5:1 men to women.

## 3. Role of Genes

In DCM, the cardiac muscle becomes thin and weakened causing the ventricular chamber to become enlarged so that the heart is unable to pump blood efficiently. For the heart to maintain cardiac output, the ventricular volume increases while contractility is reduced, producing the thin walled, dilated appearance that is characteristic of DCM. More than 30 genes have been implicated in the development of DCM through genome-wide linkage analyses, [57] candidate gene sequencing [58] and genetic association studies [59] and can be grouped into four major categories: proteins forming the myocyte cytoskeleton, sarcomeric proteins, nuclear envelope proteins and calcium homeostasis and mitochondrial function regulators [60]. Most familial DCM has an autosomal dominant inheritance pattern; however, other inheritance patterns have been identified including autosomal recessive, x-linked, mitochondrial and polygenic [59]. The most common gene in which DCM-associated variants are found is the *TTN* (Titin) gene [61], the largest known gene in humans and the third most prevalent in muscle. This gene encodes for the structural protein of the sarcomere and is prevalent in 12–25% of DCM cases (higher percentage in familial cases). Other genes commonly associated with DCM include variants in *LMNA* (Lamin A/C), *MYH7* (β-myosin heavy chain), *MYH6* (α-myosin heavy chain) and *BAG3*. Table 2 lists the genes most associated with DCM [1,2,51,57,62,63]. Several of these genes have been found to be associated with myocarditis (some with confirmed viral etiologies) in adults and children that may predispose them to developing cardiomyopathy after viral infection including *DYS* (dystrophin), *BAG3* (BAG family molecule chaperone regulator), *DSP* (desmoplakin), PKP2 (plakophilin-2), *RYR2* (ryanodine recepter-2), *SCN5A* (sodium ion channel) or *TNNI3* (cardiac troponin I) [64,65,66]. Clinical and animal model evidence exists that myocarditis can progress to DCM in susceptible individuals [44,67,68,69,70].

**Figure 2:**
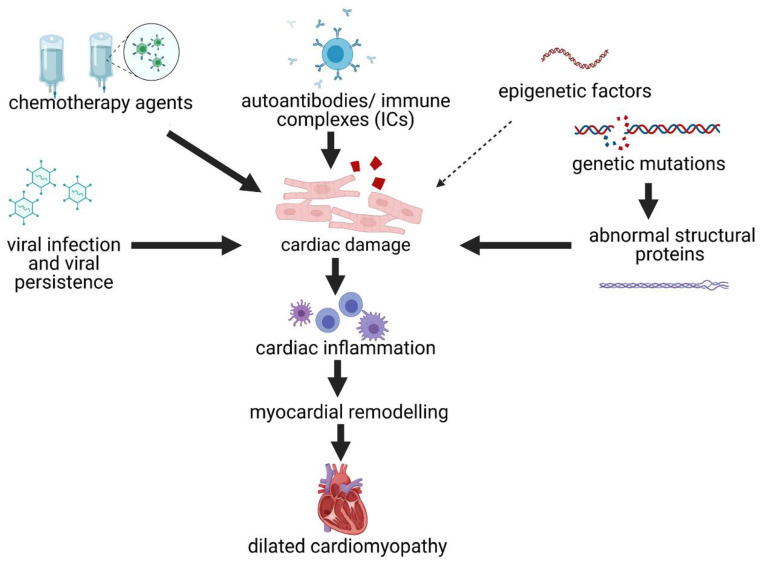
Hypothesis of interplay between genes and environment in the progression to DCM. A number of different insults can damage the myocardium initiating an immune response that leads to remodeling, fibrosis and ultimately to dilation of the ventricles and DCM. These include mutations that alter cardiac proteins, viral infections, autoantibodies, immune complexes (ICs) and toxins in the form of chemotherapy agents. (Created with BioRender.com, accessed on 31 March 2021).

The locus heterogeneity and the role of genes on disease progression is predicted to affect both sexes equally [81]. However, large scale genome-wide association studies attributing genetic profiles to disease risk have demonstrated significant sex differences [82]. These findings may be explained by the pathophysiology and pathogenesis of DCM where genetic (both rare and common variants with different modes of transmission, modifying and epigenetic factors) and environmental factors (infections or toxins that damage cardiac tissue and promote inflammation) may both contribute to disease severity, progression, age at onset and response to cardiac medication (Figure 2) [60].

Haddad et al. [83] observed sex differences in gene expression of patients with idiopathic DCM. 55 genes, mainly involved in energy metabolism and transcription regulators, were found to be differentially expressed in females compared to males (37 upregulated and 18 downregulated) while only 19 of those genes were expressed in males (13 upregulated and 7 downregulated) [83]. The dysregulated genes in males were related to myocardial contraction. Gene expression leading to ventricular remodeling were upregulated in both sexes. Sex-specific differences can be important indicators of disease progression and potentially used as diagnostic markers or as drug targets of heart failure in men and women [83]. Studies of familial DCM due to troponin T or titin mutations demonstrate a difference in phenotype in adult males (more severe) compared to females [61,84].

Next, we queried PubMed using the search terms “familial dilated cardiomyopathy” and “sex and gender differences in familial dilated cardiomyopathy” to find articles that reported genetic DCM by sex. We found 498 studies in our search; however, many studies were rejected for reasons that included the sex not being reported in the study and the study not examining genetic DCM (Figure 1). We found seven studies and two review articles that described data by sex or reported sex differences in familial/genetic DCM (Table 3). We have included one study with a pediatric population (reporting 216 adults and 129 children) because of the large number of patients in the study and because there are very few studies reporting data on genetic DCM [85]. This was the only study that had similar numbers of males and females and a similar sex ratio (Table 3), which may have been influenced by children being included in the study. The largest study comprising 492 familial DCM adult patients had a sex ratio of 1.7:1 males to females (Table 3) [52]. The average sex ratio of the five studies that had at least 300 patients with familial DCM was 1.3:1 males to females (bold in Table 3).

The sex ratio reported for familial DCM in both of the review articles was 1.5:1 males to females [89,90], but what studies the data were obtained from is not clear from the articles. The average sex ratio of familial DCM from all seven studies that we examined in this review of the literature was 1.7:1 males to females, the same as the largest study [52].

## 4. Pathogenesis of DCM

Damage to the myocardium that occurs from gene defects, viral infection or chemicals recruits immune cells to the heart in order to repair the damage. Myocardial biopsy samples or autopsies from patients with DCM have demonstrated the presence of inflammatory cell infiltrates and/or gene expression profiles indicating immune cell activation [91,92]. Known causes of inflammatory DCM include infections and autoimmunity (Figure 2) [93]. Infections, particularly viral infections like coxsackievirus B3 and parasites like *Trypanasoma cruzi* (Chagas disease), are known to cause acute cardiac inflammation or myocarditis that progresses to DCM in humans and animal models [93,94,95]. Viral infection or damaged self with adjuvant activates the complement cascade and CD11b/complement receptor (CR)3 on immune cells in animal models [96,97], and studies in humans and animal models have found that the primary factor that drives the progression from myocarditis to DCM is complement activation [97,98]. TLR4 on mast cells and macrophages increase the proinflammatory and profibrotic cytokine IL-1β that promotes fibrosis [99,100,101]. A T helper 1 (Th1)-type immune response associated with interferon (IFN)-γ prevents remodeling and progression to DCM after myocarditis, while Th2- (IL-4 and IL-33-associated cytokines) and Th17-type (IL-17A and IL-6-associated cytokines) immune responses promote remodeling, fibrosis and DCM in mouse models and human studies [68,102,103,104,105,106,107,108,109]. Cardiac specific autoantibodies that are associated with viral and autoimmune myocarditis are able to deposit as immune complexes (ICs) with complement on cardiac tissue including the pericardium and further promote damage, inflammation and fibrosis [97,100,110,111,112,113,114]. Remodeling genes are upregulated in the heart during acute viral myocarditis during the peak of inflammation (day 10 after infection), but fibrosis only appears in the heart several weeks later after inflammation has largely subsided (day 35 after infection) [101]. Thus, fibrotic scar tissue eventually replaces the damaged tissue, leading to stiffening of the heart, dilatation and progression to heart failure in susceptible individuals (Figure 2).

DCM also occurs secondary to chemotherapy for cancer. The number of patients diagnosed with DCM secondary to antiblastic drugs is rising, due to the increasing age of the general population and the increase in survival times, attributed to the success of earlier diagnosis and new therapies [115]. DCM can be the final phase of a pathological process that is usually defined more broadly as chemotherapy-related cardiac dysfunction (CRCD). CRCD is reported to affect up to 10% of cancer survivors [116]. There are two types of cardiotoxicity- reversible myocardial dysfunction that is independent of the drug dose and irreversible myocardial damage that occurs due to a cumulative dose of the drug. The exact mechanism leading to DCM is unclear and it is believed that damage occurs through the generation of reactive oxygen species, accumulation of anthracycline metabolites that disrupt sarcomere structure and function and mitochondrial dysfunction [117,118]. These processes lead to cardiac remodeling and ultimately, dilation of the heart giving rise to DCM. Whether inflammation is involved in this process is less clear because cardiac biopsies are not routinely obtained from these patients and there are not good animal models to replicate the pathogenesis of disease.

## 5. Sex Differences in Genetic DCM

As mentioned previously, the locus heterogeneity and the role of genes on disease progression is predicted to affect both sexes equally yet sex differences exist in genetic DCM. So, why does DCM from genetic causes occur more often in men when it would be expected to occur at a similar rate in men and women? First, it is important to understand that underlying sex differences in tissue physiology influence cardiac function in a sex-specific manner even in healthy, normal hearts and influence how the heart and immune system respond to injury or infection. Sex hormone receptors such as ERα/β, AR and aromatase, the enzyme that converts androgens to estrogens, are expressed both on/in cardiac tissues as well as on/in cells of the immune system [8,10,45,46,119]. Estrogen and androgen receptors are expressed within vascular endothelial cells, vascular smooth muscle cells, cardiac fibroblasts and cardiomyocytes in humans and rodents [8,10,45,46,119]. Additionally, sex hormone receptors may be expressed at different levels by sex; females express higher levels of ERs in their arteries for example and men have higher levels of testosterone in their heart than females [46,47,119]. These underlying sex differences contribute to differences in cardiac physiology by sex. Even though there were only a handful of studies of familial/genetic DCM that reported the number of men and women in their study that we were able to find in our PubMed search (Table 3), these studies reported a higher number of men with DCM compared to women similar to studies of DCM from all causes (Table 1). More familial studies of patients with DCM need to be conducted to determine whether a sex ratio of 1.5:1 to 1.7:1 male to female remains consistent and whether a greater sex difference is observed with general DCM (2.5:1 male to female) or whether there is essentially no difference in the sex ratio between the two categories of DCM (i.e., all causes/non-genetic vs. genetic) once more patients are examined. A meta-analysis needs to be conducted once more studies have been performed to determine the statistical significance of the sex ratio and if the ratio is lower in genetic cases of DCM when compared to non-genetic cases. A couple of studies of genetic DCM have reported similar numbers of males and females affected but adverse events occurred earlier in male than female carriers indicating a clear sex-difference [61,120].

The second point is that physical damage to the heart that occurs from genetic causes (i.e., pathogenic variants in titin), damage from toxins (i.e., chemotherapy agents like doxorubicin) and/or infections (i.e., coxsackievirus) is most likely going to activate an immune response to the damage and if it does, that immune response will be sex specific. Sex hormone receptors are located on/in many cells of the immune system including T cells, B cells, monocytes, macrophages, dendritic cells (DCs) and mast cells (MCs) in humans and rodents [55,120]. Importantly, only monocyte/macrophages and mast cells have both nuclear and membrane ERs and the AR [55,120]. MCs are critically important cells that drive sex-specific differences in response to cardiac damage by acting as antigen presenting cells (APCs), degranulating in response to infections or toxins/chemicals and initiating immune responses through TLRs and other innate immune receptors [112,121,122,123]. Mast cells also play a central role in mediating cardiac remodeling that leads to DCM by releasing other factors like enzymes such as serpin A3n (α1-antichymotrypsin) that cleave/activate profibrotic cytokines such as interleukin (IL)-1β and TGF-β1 to their active form and MMPs [101]. 17β-estradiol has been found to mediate a protective effect in response to cardiac damage, regardless of the cause of the damage, by reducing apoptosis in cardiac myocytes, reactive oxygen species-induced cardiac damage and preventing cardiac hypertrophy and fibrosis [120,121,122,123]. Estrogen has been found to decrease proinflammatory TLR4-induced cytokine expression of IL-1β, IL-6, TNFα from monocyte/macrophages [124]. TLR4 and these proinflammatory cytokines have been found to promote remodeling and fibrosis that leads to DCM in patients and animal models of myocarditis [101,125], which occurs more often in males than females [12]. Estrogen inhibits neutrophil infiltration, oxidant stress and necrosis in an ER-dependent manner following ischemia/reperfusion. TNFα levels increase in ovariectomized rats that have reduced circulating estrogen levels when subjected to ischemia that reverses when estrogen is restored [126]. Aside from inflammation that comes into the heart in response to cardiac injury (i.e., mutation altered protein structure, viral damage, toxin damage with chemotherapy), sex hormones alter cardiac function by binding to androgen and estrogen receptors on cardiac vascular endothelial cells, smooth muscle cells, fibroblasts and myocytes [13,98,110]. ERβ has been found to inhibit reactive oxygen species-mediated NF-қB inflammation [127]. In contrast, testosterone activated the NF-қB pathway leading to an increase in inflammation and hypertrophy [12,122,123,128].

## 6. Genetics and Environment

Genetic variants that alter the structure of proteins in the heart increase the risk for developing cardiomyopathy/DCM. However, some variants may not appear until an environmental pressure such as an infection or toxin/chemical inflicts damage, which may increase the risk for progression to chronic cardiomyopathy or more severe disease and heart failure in these individuals. This may partly explain why common viral infections like coxsackieviruses lead to cardiac inflammation and heart failure in only a small number of individuals compared to the nearly 100% infectivity rate for the virus. Similarly, genetic background may increase the risk for cardiomyopathy at lower chemotherapy (i.e., anthracycline) doses than for individuals without mutations. If an interplay occurs between environmental factors and genetic variations, then gene variants could be used to predict risk of developing cardiomyopathy following cardiac damage. These patients might be sensitive to anthracyclines independent of the dose given [129]. Early identification of cardiac damage would be important to intervene to prevent remodeling and progression to DCM and heart failure. Sera biomarkers like troponins and NTpro-BNP are routinely monitored to identify cardiac damage, yet they may only be effective at detecting more severe cardiac damage as occurs with myocardial infarct. They are often less effective at detecting mild myocardial damage from a viral infection, for example, that can none-the-less still progress to DCM. There remains a need to find more sensitive methods to detect risk of progression to DCM.

## 7. Limitations

Limitations to the study include using only PubMed to search for articles. Additionally, we searched PubMed for articles using “sex and gender” as terms, but we do not discuss the influence of gender on the progression of DCM. We did not conduct a formal ‘meta-analysis’ to determine whether the sex ratio obtained from the manuscripts reporting men and women with idiopathic or non-genetic DCM were significantly different from those with genetic DCM, primarily because there are so few genetic DCM studies reporting data by sex. We also did not examine or discuss sex differences in pediatric DCM. Finally, although genetic defects and environmental factors like viruses and chemicals (chemotherapy agents) are able to cause DCM, the cause of most cases seen in the clinic remain unknown or idiopathic.

## 8. Conclusions and Future Directions

Sex differences occur in DCM for all causes including familial/genetic, with more males developing disease than females. However, there is a need for clinical and basic research studies that investigate the mechanisms behind the sex differences. Additionally, it is critical that studies report data according to sex (and age) in order to better understand the progression of disease in both sexes. This will enable biomarker discovery and therapeutic targets in the future and personalized management of patients. Based on what is known about the pathogenesis of DCM, we postulate that if biopsies were taken from genetic and idiopathic cases of DCM there would be evidence of inflammation, fibrosis and remodeling of the damaged tissue similar to the pathological changes that have been observed during viral myocarditis/acute DCM. Yet there are still many unanswered questions and further research is needed to deepen our understanding of the pathogenesis of disease.

## Figures and Tables

**Table 1:** Sex ratio in studies of DCM ^a,b^.

Year of Publication	Patients (*n*)	Male:Female (*n*)	Sex Ratio (M:F)	Mean Age of Patients	Additional Information	References
1949	35	25:10	2.5:1	–		[27]
1968	22	13:9	1.4:1	–	Coxsackievirus heart disease	[28]
1980	164	109:55	1.9:1	–	Coxsackievirus myocarditis progressing to DCM	[29]
1984	41	27:14	1.9:1	–	DCM	[30]
1987	72	59:13	4.5:1	50 ± 15	DCM	[31]
1990	201	163:38	4.2:1	48 ± 11	DCM	[32]
1992	225	163:62	2.6:1	41 ± 12.3	DCM	[33]
1993	303	238:65	3.6:1		Idiopathic DCM	[34]
1994	128	68:60	1.1:1	59	DCM	[35]
1995	441	309:132	2.3:1	43 ± 13	DCM	[36]
1996	144	118:26	4.5:1	39 ± 10.4	Nonischemic DCM	[37]
2000	131	108:23	4.7:1	52 ± 12	DCM	[38]
2004	458	326:132	2.4:1	58.3	Nonischemic DCM	[39]
2004	56	42:14	3:1	50.3 ± 2.2	DCM	[40]
2005	20	14:6	2.3:1	46.5 ± 10	Recent onset CM	[10]
2008	54	38:16	2.3:1	–	DCM in elderly (65–83 years of age)	[41]
2010	43	29:13	2.2:1	–	Idiopathic DCM with new onset HF	[42]
2010	115	100:35	1.5:1	–	DCM	[43]
2011	373	230:143	1.6:1	45 ± 14		[44]
2012	95	52:43	1.2:1	–	95 DCM patients vs. 95 healthy subjects	[45]
**2013 ^c^**	**603**	**440:163**	**2.7:1**	–	**Idiopathic DCM**	[46]
2013	96	66:30	2.2:1	53 ± 11.6	DCM	[47]
2014	373	230:143	1.6:1	45 ± 14	DCM	[48]
**2014**	**853**	**614:239**	**2.5:1**	**45 ± 15**	**Idiopathic DCM**	[49]
2015	213	128:85	1.5:1	–	DCM	[50]
2015	639	405:212	1.9:1	–	DCM	[51]
**2015**	**16,091**	**11,059:5032**	**2.2:1**	**48.3 ± 12.6**	**DCM UNOS Database**	[52]
**2018**	**881**	**591:290**	**2:1**	**52 ± 15**	**DCM**	[53]
2018	52	40:12	3.3:1	57.2 ± 7	Non-ischemic DCM	[54]
**2018**	**1260**	**935:325**	**2.8:1**	**–**	**CM Registry-DCM**	**[55]**
2019	35	24:11	2.1:1	–	DCM with/without PH	[56]

^a^ Abbreviations: CM Registry, cardiomyopathy registry for the EURObservational Research Programme (used DCM data); DCM, dilated cardiomyopathy; F, female; HF, heart failure; M, male; PH, pulmonary hypertension. ^b^ Some overlap exists in these studies which include genetic and other causes of DCM. ^c^ Five studies with largest number of patients (over 500) are highlighted in bold in the table.

**Table 2:** Most common genes involved in DCM (in descending order).

Gene	Encoding Protein	Function of the Protein	References
*TTN*	Titin	Forms the structure of the sarcomere	[61]
*LMNA*	Lamin A/C	Nuclear membrane envelope	[71]
*MYH7*	Beta-myosin heavy chain	Sarcomere	[57]
*MYH6*	Alpha myosin heavy chain	Sarcomere	[57]
*BAG3*	BAG family molecular chaperone regulator	Chaperone-assisted selective autophagy	[72,73]
*MYPN*	Myopalladin	Z-disc in the sarcomere	[74]
*DSP*	Desmoplakin	Desmosome	[75]
*FLNC*	Filamin C	Functions at Z discs	[76]
*RBM20*	RNA- binding protein 20	Spliceosome, RNA-binding protein	[77]
*TTNT2*	Cardiac Troponin T	Sarcomere	[57]
*SCN5A*	Sodium ion channel	Ion channel	[78,79]
*TTNC1*	Cardiac Troponin C	Sarcomere	[57]
*TTNI3*	Cardiac Troponin I	Sarcomere	[57]
*TPM1*	Alpha- Tropomyosin	Sarcomere	[58,80]

**Table 3:** Sex ratio in studies of familial DCM ^a^.

Year of Publication	Patients (*n*)	Male:Female	Sex Ratio (M:F)	Mean Age of Patients	Mode of Inheritance	References
1989	45	33:12	2.7:1	54		[26]
**1999 ^b^**	**345**	**169:176**	**0.97:1**	**Adults (38.3 ± 14) and children (<16 years of age)**	**AD**	[85]
**2006**	**304**	**173:131**	**1.3:1**	–	**AD (most common)**	[86]
**2012**	**312**	**208:104**	**2:1**	**48.6 ± 13.0**		[61]
**2013**	**418**	**233:185**	**1.3:1**	**46 ± 13**		[87]
**2015**	**492**	**311:181**	**1.7:1**	**42.1 ± 13.1**		[52]
2017	72	48:24	2:1	34	AD (TTN +/−)	[88]

^a^ Abbreviations: AD, autosomal dominant; AR, autosomal recessive; F, female; M, male; TTN, titin. ^b^ Five studies with largest number of patients (over 300) are highlighted in bold in the table.

## Data Availability

All data are available within the manuscript.
